# Combination of LBP Bin and Histogram Selections for Color Texture Classification

**DOI:** 10.3390/jimaging6060053

**Published:** 2020-06-23

**Authors:** Alice Porebski, Vinh Truong Hoang, Nicolas Vandenbroucke, Denis Hamad

**Affiliations:** 1 LISIC laboratory, Université du Littoral Côte d’Opale, 50 rue Ferdinand Buisson, 62228 Calais CEDEX, France; nicolas.vandenbroucke@lisic.univ-littoral.fr (N.V.); denis.hamad@lisic.univ-littoral.fr (D.H.); 2 Faculty of Information Technology, Ho Chi Minh City Open University, 97 Vo Van Tan, District 3, 700000 Ho Chi Minh City, Vietnam; vinh.th@ou.edu.vn

**Keywords:** texture classification, color spaces, feature selection, local binary pattern descriptor

## Abstract

LBP (Local Binary Pattern) is a very popular texture descriptor largely used in computer vision. In most applications, LBP histograms are exploited as texture features leading to a high dimensional feature space, especially for color texture classification problems. In the past few years, different solutions were proposed to reduce the dimension of the feature space based on the LBP histogram. Most of these approaches apply feature selection methods in order to find the most discriminative bins. Recently another strategy proposed selecting the most discriminant LBP histograms in their entirety. This paper tends to improve on these previous approaches, and presents a combination of LBP bin and histogram selections, where a histogram ranking method is applied before processing a bin selection procedure. The proposed approach is evaluated on five benchmark image databases and the obtained results show the effectiveness of the combination of LBP bin and histogram selections which outperforms the simple LBP bin and LBP histogram selection approaches when they are applied independently.

## 1. Introduction

Texture analysis is one of the major topics in the field of computer vision and has many important applications including face recognition, object detection, image filtering, segmentation, and content-based access to image databases [1]. Texture classification can be defined as a task that assigns a texture into one of a set of predefined categories. This step requires an efficient descriptor in order to represent and discriminate the different texture classes. In past decades, texture analysis was extensively studied and a wide variety of texture representations were proposed [2]. Among these approaches, the Local Binary Pattern (LBP) descriptor proposed by Ojala et al. is known as one of the most successful statistical approaches due to its efficiency, robustness against illumination intensity changes, and relative fast calculation [3]. It was successfully applied to numerous applications as diverse as texture classification. To encode LBP, the gray level of each pixel is compared with those of its neighbors and the results of these comparisons are then weighted and summed. The obtained texture descriptor is the LBP histogram whose size depends on the number of neighbors. Since 2002, several extensions of LBP to color were then proposed to take advantage of all the color texture information contained in an image [4]. Considering a particular color space, the LBP descriptor is thus applied on each color component independently or on pairs of color components jointly, leading to several LBP histograms for characterizing a color texture.

To improve the classification performance, many approaches were proposed to reduce the dimension of the feature space based on the LBP histogram(s) [4]. These approaches follow four strategies.

(1) To obtain more discriminative, robust and compact LBP-based features, the first strategy consists of identifying the most informative pattern groups based on some rules or on the predefinition of patterns of interest. The uniform LBP operator, where a reduced number of discriminant bins are *a priori* chosen among all the available ones, is an example of predefinied compact LBP [5].
(2) The second strategy is based on feature extraction approaches, which project features into a new feature space, with a lower dimensionality, where the new constructed features are usually combinations of the original features. Chan et al. use a linear discriminant analysis to project high-dimensional color LBP bins into a discriminant space [6]. Banerji et al. apply Principal Component Analysis (PCA) to reduce the feature dimensionality of the concatenating LBP features extracted from different color spaces. Zhao et al. compare different dimensionality reduction methods on LBP features, e.g., PCA, kernel PCA and Laplacian PCA [7]. Hussain et al. exploit the complementarity of three sets of features, including LBP, and applies partial least squares for improving their object class recognition approach [8].
(3) The third strategy consists of applying feature selection methods in order to find the most discriminative patterns [9]. Smith and Windeatt apply the fast correlation-based filtering algorithm [10] to select the LBP patterns that are the most correlated with the target class [11]. This algorithm starts with the full set of features, calculates dependences of features thanks to symmetrical uncertainty, and finds the best subset using backward selection technique with sequential search strategy. Lahdenoja et al. define a discrimination concept of symmetry for uniform patterns to reduce the feature dimensionality [12]. In this approach, the patterns with a higher level of symmetry are shown to have more discriminative power. Maturana et al. use an algorithm based on Fisher-like class separability criterion to select the neighbors used in the computation of LBP [13]. Liao et al. introduce Dominant Local Binary patterns (DLBP) which consider the most frequently occurred patterns in a texture image [14]. To compute the DLBP feature vectors from an input image, a pattern histogram which considers all the patterns in the input image is constructed and the histogram bins are sorted in descending order. The occurrence frequencies corresponding to the most frequently occurred patterns in the input image are served as the feature vectors. Guo et al. propose a Fisher separation criterion to learn the most reliable and robust patterns by using intra-class and inter-class distances [15]. This approach, which proposes to learn the most reliable and robust dominant bins by considering intra-class similarity and inter-class dissimilarity, is very interesting since it outperforms Ojala’s uniform LBP operator and Liao’s DLBP in the experiments on three texture databases. It has also been recently extended to the multi color space domain [16].
(4) A fourth strategy to reduce the dimension of the feature space based on LBP histograms was proposed by Porebski et al. in 2013 [17]. In this approach, the most discriminant LBP histograms are selected in their entirety out of the different LBP histograms extracted from a color texture. It fundamentally differs from all the previous approaches which select the bins of the LBP histograms or project them into a discriminant space. Several scores were proposed in the literature to evaluate the relevance of histograms: the Intra-Class Similarity score (ICS-score), proposed by Porebski et al. [17], which is based on an intra-class similarity measure; the Adapted Supervised Laplacian score (ASL-score) and Adapted Laplacian score (AL-score), proposed by Kalakech et al. [18,19], which evaluates the relevance of the histograms using the local properties of the image data; the Simba-2 score, proposed by Mouhajid et al. [20], which is based on the hypothesis margin and the $\chi^{2}$ distance; and the Sparse Adapted Supervised Laplacian score (SpASL-score) [21], which is based on the ASL-score and a sparse representation. The LBP histogram selection approach using ICS or ASL scores was recently extended to the multi color space domain and showed its relevance compared to the bin selection approach proposed by Guo [16].

In this paper, we propose improving this last selection strategy that selects discriminant LBP histograms in their entirety. Indeed, it is clear that not all bins of the selected histograms are meaningful for modelling the characteristics of textures. As it selects the most discriminating histograms and filter out the rest, we think that it might have some redundant bins in the selected histograms and a loss of some meaningful bins of the discarded histograms. That is why we propose introducing the combination of LBP bin and histogram selections, where a histogram ranking method is applied before processing a bin selection procedure. Two minor and one major contributions are thus proposed in this paper:

- For the first minor contribution, the LBP histogram selection approach proposed in [21], using the SpASL-score, is extended to the multi color space domain to define the Sparse Multi Color Space Histogram Selection (Sparse-MCSHS).
- We then propose comparing this Sparse-MCSHS approach with a multi color space bin selection approach, also based on a sparse representation. For this purpose, the sparsity score proposed by Liu [22] is used for selecting the most discriminant bins of LBP histograms extracted from images coded in several color spaces, leading to the Sparse Multi Color Space Bin Selection (Sparse-MCSBS) approach. This second minor contribution corresponds to the MCSBS approach proposed in [16] that was extended to a sparse representation.
- These two approaches, Sparse-MCSHS and Sparse-MCSBS, are then combined to form the Sparse-MCSHBS approach (Sparse Multi Color Space Histogram and Bin Selection). This combination of bin and histogram selections represents the main contribution of the paper since such a combination for selecting relevant LBP bins was never previously proposed.

In Section 2, the color LBP histograms used to represent color textures are first described. The proposed Sparse-MCSHS and Sparse-MCSBS approaches are then presented in Section 3. The Section 4 introduces the combination of bin and histogram selections and finally experiments are carried out on benchmark texture databases in Section 5.

## 2. Color LBP Histograms

The LBP operator is widely applied to define texture features for classification of gray level images due to its inherent simplicity and robustness [3]. It transforms an image by thresholding the *P* neighbor levels of each pixel and coding the result as a binary number (where *P* is the number of neighboring pixels). Usually, the histogram of this LBP image is then computed for texture analysis and many authors take an interest in reduction of the $2^{P}$-dimensional LBP histograms in order to improve texture classification performance [4].

The original LBP computation, based on gray level images, was then extended to multiple variants [23] and also to color since it was demonstrated that color information is a highly discriminant visual cue to represent the texture, especially in natural textures [24,25,26,27,28]. In the literature, the extension of LBP to color follows four different strategies: one in which informations of color and texture are separately analyzed (1), and the three others where color and texture are jointly considered during the computation of the LBP descriptor (2-3-4) [29,30,31].

(1) In the first strategy, the original LBP operator is computed from the luminance image and combined with color features. For example, Mäenpää or Ning proposed to represent the color texture by concatenating the 3D color histogram of the color image and the LBP histogram of the corresponding luminance image [31,32]. Cusano et al. propose a texture descriptor which combines a luminance LBP histogram with color features based on the local color contrast [33].
(2) The second strategy is a marginal approach that consists of applying the original LBP operator independently on each of the three components of the color image, without considering the spatial interactions between the levels of two different color components. The texture descriptor is obtained by concatenating the three resulting LBP histograms. This within component strategy was applied by several authors [34,35,36,37,38].
(3) The third strategy consists of taking into account the spatial interactions within and between color components. To describe color texture, Opponent Color LBP (OCLBP) was defined [31]. For this purpose, the LBP operator is applied on each pixel and for each pair of components denoted $\left(C_{k},C_{k}^{\prime }\right)$, $k,k^{\prime }\in \left\{1,2,3\right\}$. In this definition, opposing pairs such as $\left(C_{1},C_{2}\right)$ and $\left(C_{2},C_{1}\right)$ are considering to be highly redundant, and so, one of each pair is taken into consideration in the analysis. This leads to characterize a texture with only six histograms pairs $(\left(C_{1},C_{1}\right)$, $\left(C_{2},C_{2}\right)$, $\left(C_{3},C_{3}\right)$, $\left(C_{1},C_{2}\right)$, $\left(C_{1},C_{3}\right)$, $\left(C_{2},C_{3})\right)$ out of the nine available ones. However, these *a priori* chosen six histograms are not always the most relevant according to the different considered data sets [17] and it is preferable to consider the Extended Opponent Color LBP (EOCLBP). This way to describe the color textures with LBP was proposed by Pietikäinen in 2002 [37]. It consists of taking into account each color component independently and each possible pair of color components, leading to nine different histograms: three within-component $\left(\left(C_{1},C_{1}\right),\left(C_{2},C_{2}\right),\left(C_{3},C_{3}\right)\right)$ and six between-component $(\left(C_{1},C_{2}\right)$, $\left(C_{2},C_{1}\right)$, $\left(C_{1},C_{3}\right)$, $\left(C_{3},C_{1}\right)$, $\left(C_{2},C_{3}\right)$, $\left(C_{3},C_{2})\right)$ LBP histograms. These nine histograms are finally concatenated so that a color texture image is represented in a $\left(9\times 2^{P}\right)$-dimensional feature space. The OCLBP and EOCLBP have often been considered to classify color texture images [6,17,18,31,39,40]. Recently, Lee et al. propose another color LBP variant for face recognition tasks, the local color vector binary pattern [41]. In the proposed approach, each color texture image is characterized by the color norm pattern and the color angular patterns via LBP texture operation.
(4) The fourth strategy consists of analyzing the spatial interactions between the colors of the neighboring pixels based on the consideration of an order relation between colors following a vectorial approach. Instead of comparing the color components of pixels, Porebski et al. represent the color of pixels by a vector and compare the color vectors of the neighboring pixels with the color vector of the central one [42]. They use a partial color order relation based on the Euclidean distance for comparing the rank of color. As a result, a single color LBP histogram is obtained instead of the 6 or 9 provided by OCLBP or EOCLBP, respectively [17,18]. Another possible way consists of defining a suitable total ordering in the color space. This strategy was investigated by Ledoux et al. who propose the Mixed Color Order LBP (MCOLBP) [43]. Finally, in order to give a single code by color LBP, the quaternion representation can be considered. Quaternion is shown to be an efficient mathematical tool for representing color images based on a hypercomplex representation [44]. Lan et al. have thus proposed the Quaternionic Local Binary Pattern (QLBP) that makes use of quaternion to represent each pixel color by a complex number including all color components at one time. Under this representation, the dimension of QLBP is equal to the dimension of a grayscale LBP. QLBP was applied for person re-identification problems by Lan and Chahla in [45,46] and was then extended in [47] for color image classification.

Among the different extension strategies of LBP to color, MCOLBP and QLBP have the advantage of providing a texture descriptor whose dimension is equal to gray level LBP histogram, and thus a low computation time. However, the classification accuracies obtained with these descriptors on two benchmark texture databases are not as high as those obtained with OCLBP [43,48]. That is the reason we propose in this paper to consider the color LBP coming from the third strategy, and particularly the EOCLBP, where no *a priori* choice of the most discriminant LBP histograms is done. To reduce the dimension of this $\left(9\times 2^{P}\right)$-dimensional LBP-based feature space, three selection procedures based on a sparse representation are presented and compared to automatically select the most discriminant features: the Sparse-MCSHS and Sparse-MCSBS approaches, described in the next section, and the combination of bin and histogram selections presented in Section 4.

## 3. Sparse-MCSHS and Sparse-MCSBS Approaches

Many authors have lead studies about the choice of color space for different applications: machine vision, face recognition, texture analysis, etc. [49,50,51]. Indeed, there exist numerous color spaces and it is proved that the color space choice impacts the results [52]. In the framework of color texture classification, many authors try to determine the “best” color space in order to improve the results of their proposed classification approach. However, it was shown that it is difficult to *a priori* determine the best color space suited to all applications of color texture classification [16]. For this reason, an alternative approach emerged: it consists of simultaneously exploiting the properties of several color spaces. Three main strategies are proposed in the literature:

- Color space fusion, which involves fusing the results from several classifiers, each one operating in a different color space [53,54,55,56],
- Color space selection, which consists of selecting the most well suited color spaces which are based on some specific quality criteria [57,58,59,60],
- Color texture feature selection that evaluates the texture features over different color spaces and selects the set of features that provide the best discrimination between the different textures classed by using a supervised feature selection approach [34,61,62,63,64].

There exists no study that compares the performance of these color space combination strategies and it could be a great prospect to compare them. In this paper, we propose using the color texture feature selection strategy to compare and combine the approaches of LBP histogram selection and LBP bin selection in a multi color space framework.

### 3.1. Considered Color Spaces

As explained previously, there exist numerous color spaces. These color spaces take into account different physical, physiologic and psycho-visual properties, and can be classified into four families [60]. $N_{S}=9$ color spaces are here considered for experiments:

- RGB and rgb, which belong to the primary space family,
- $YC_{b}C_{r}$ and (wb,rg,by), which are luminance-chrominance spaces,
- $I_{1}I_{2}I_{3}$, which is an independent color component space,
- *HSV*, *HSI*, *HLS* and *I-HLS*, which belong to the perceptual space family.

These nine color spaces were chosen since they do not require to know illumination and image acquisition conditions, contrary to CIE $L^{*}a^{*}b^{*}$ or $L^{*}u^{*}v^{*}$ color spaces for instance. They are also representative of the four different color space families, even if a majority of perceptual spaces were chosen because these spaces are known to obtain good classification accuracies [63,65].

### 3.2. Candidate Color Texture Descriptors

To compute the color LBP histograms or bins that are candidate for the selection, each image is first coded in each of the $N_{S}=9$ color spaces previously presented. Then, for each of this color space, the $\delta_{max}=9$ different LBP histograms of the EOCLBP descriptor are computed from the so-coded images. A color texture is thus represented by $\delta_{max}\times N_{S}=9\times 9=81$ candidate LBP histograms. When the number of bins *Q* is equal to 256 for each histogram (with P=8, $Q=2^{P}$), the total number of candidate LBP bins is $Q\times \delta_{max}\times N_{S}=256\times 9\times 9=$ 20,736 bins. Once the color texture descriptors have been computed, the most discriminant features are selected out of the available candidate LBP-based features (histograms or bins) in a supervised context in order to build a feature subspace with a reduced dimension, in which color textures are represented.

To evaluate the relevance of the feature subspaces, different models are proposed in the supervised context where samples with known class labels are available for the learning stage [66]. Wrapper models are defined by a feature selection procedure that uses the classification accuracy as discrimination power of a feature subspace. It gives good results and easily determines the dimension of the feature subspace by searching the highest classification rate but involves an important learning time and classifier-dependent results. On the contrary, filter models are built by using feature selection procedures that evaluate the discrimination power of different candidate feature subspaces without classifying the images. They are less time consuming but suffer to the difficulty to determine the dimension of the feature subspace to be selected. To obtain a good compromise between dimension selection, computation time and classification result, embedded models are preferred [67]. These approaches combine a filter model to determine the most discriminating feature subspaces at different dimensions and a wrapper model to determine the dimension of the selected subspace [68]. In this framework, wrapper and embedded selection approaches require to split up the initial dataset available for the learning stage in order to build a training and a validation subset. The classification stage is thus operated in the selected feature subspace with a testing subset.

### 3.3. Sparse-MCSHS Approach

The Multi Color Space Histogram Selection (MCSHS) approach analyzes LBP histograms computed from texture images coded into several color spaces [16]. Indeed, rather than looking for the best color space, these approaches first compute LBP histograms from several color spaces and then selects, out of the different candidate LBP histograms, those which are the most discriminant for the considered application in a supervised context.

**Figure 1:**
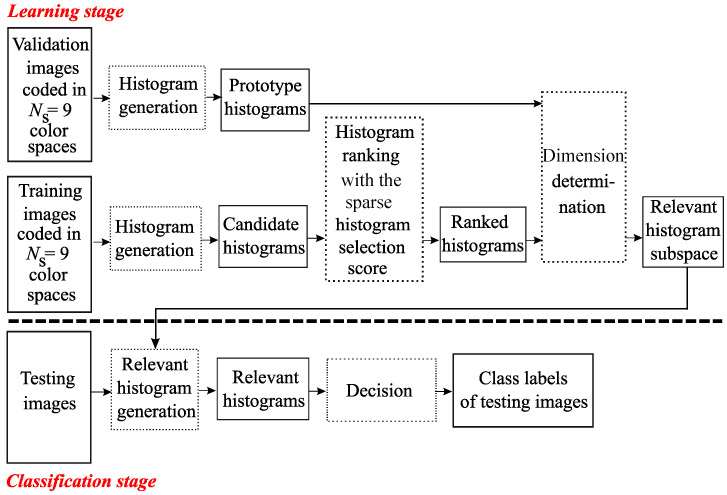
The Sparse-MCSHS approach.

MCSHS is an embedded histogram selection approach whose flow chart is represented by Figure 1. During the learning stage, candidate histograms are generated from training images. Then, the proposed histogram selection procedure uses a ranking algorithm. The selection is based on the histogram score evaluated for each of the $\delta_{max}\times N_{S}=81$ available histograms. In previous works, several scores were proposed in the literature to evaluate the relevance of histograms: the Intra-Class Similarity score (ICS-score), proposed by Porebski et al. [17], which is based on an intra-class similarity measure; the Adapted Supervised Laplacian score (ASL-score), proposed by Kalakech et al. [18], which evaluates the relevance of histograms using the local properties of the image data; the Simba-2 score, proposed by Mouhajid et al. [20], which is based on the hypothesis margin and the $\chi^{2}$ distance; and the Sparse Adapted Supervised Laplacian score (SpASL-score) [21]. Only the two first scores were applied in a multi color space framework. In this paper, we propose extending the multi color space domain with the LBP histogram selection approach using the SpASL-score.

The SpASL-score is an extension of the ASL-score proposed by Kalakech et al. [18]. The ASL-score is based on the Jeffrey distance and a similarity matrix which is deduced from the class labels with hard values 0 or 1. The SpASL-score uses a sparse representation to build a soft similarity matrix that takes float values between 0 and 1. Such a value measures the similarity in a subtle way, instead of being binary with just two values 0 and 1. This lead to a more powerful discriminating information [21].

Once the score has been computed for each of the $\delta_{max}\times N_{S}=81$ candidate prototype histograms, a ranking is performed. The next step then consists of determining the dimension of the relevant histogram subspace. For this purpose, the candidate subspaces—made up, at the first step of the procedure, of the histogram with the best score, at the second step, of the two first ranked histograms and so on—are evaluated (see Figure 1). The evaluation function at different dimensions and the stopping criterion of the histogram selection procedure are based on the classification accuracy. In this work, accuracy is measured by using the nearest neighbor classifier associated with the L1 distance as a similarity measure because it does not require any parameter to be adjusted during the learning stage. This evaluation leads to consider during the learning stage a subset different from the training subset, i.e., the validation images. For this purpose, the classifier operates in each candidate subspace in order to classify the validation images represented by their prototype histograms. The selected subspace, whose dimension is $\hat{D}=\hat{\delta }\times Q$, is the one which maximizes the rate $R_{\delta }$ of well-classified validation images:

(1) $$\hat{\delta }=\underset{1\leq \delta \leq \delta_{max}\times N_{S}}{\mathrm{argmax}}R_{\delta }.$$

During the classification stage, textures of testing images are represented in the so-selected relevant histogram subspace in order to determine their class labels.

### 3.4. Sparse-MCSBS Approach

**Figure 2:**
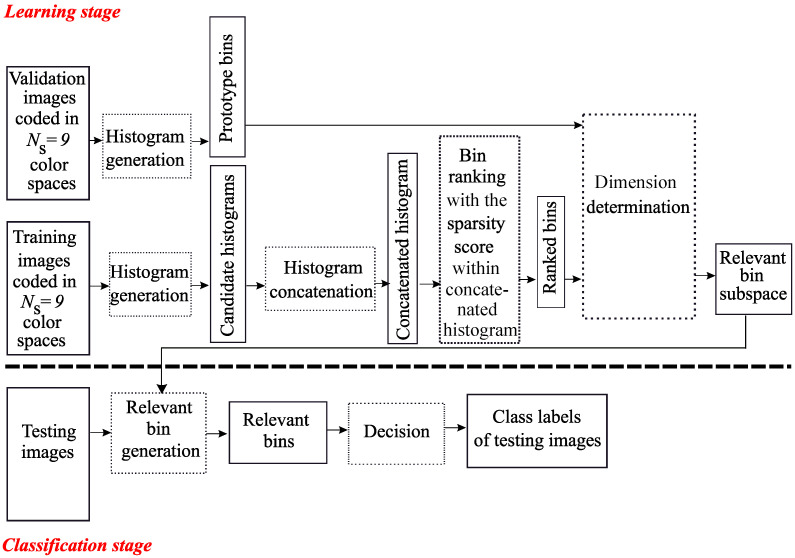
The Sparse-MCSBS approach.

Like MCSHS, the Multi Color Space Bin Selection (MCSBS) approach analyzes LBP histograms computed from texture images coded into several color spaces. Instead of selecting the most discriminating histograms, MCSBS selects the most discriminating bins of these histograms. A first approach of MCSBS was proposed by Porebski et al. in 2018 [16]: it is an extension to the multi color space domain of the bin selection method proposed in 2010 by Guo et al. for gray level image analysis [15]. In this paper, we consider that each bin of an histogram corresponds to a feature of a vector, and we propose applying an embedded model for selecting the most discriminant bins. In the MCSHS approach, the SpASL-score, based on sparse representation, was considered to select relevant histograms. In the MCSBS approach, we also propose considering a score based on sparse representation. Among the effective supervised ranking scores, the supervised sparsity score outperforms other scores as shown in [22]. So we propose extending this score for selecting LBP histogram bins in the multi color space domain. The flow chart of the proposed MCSBS approach is represented by Figure 2.

During the learning stage, candidate histograms are generated from training images and concatenated to form a vector with $Q\times \delta_{max}\times N_{S}=$ 20,736 features. The sparsity score proposed by Liu is then computed for each feature [22]. The ranked bins are obtained by sorting all bins according to their score in ascending order.

**Figure 3:**
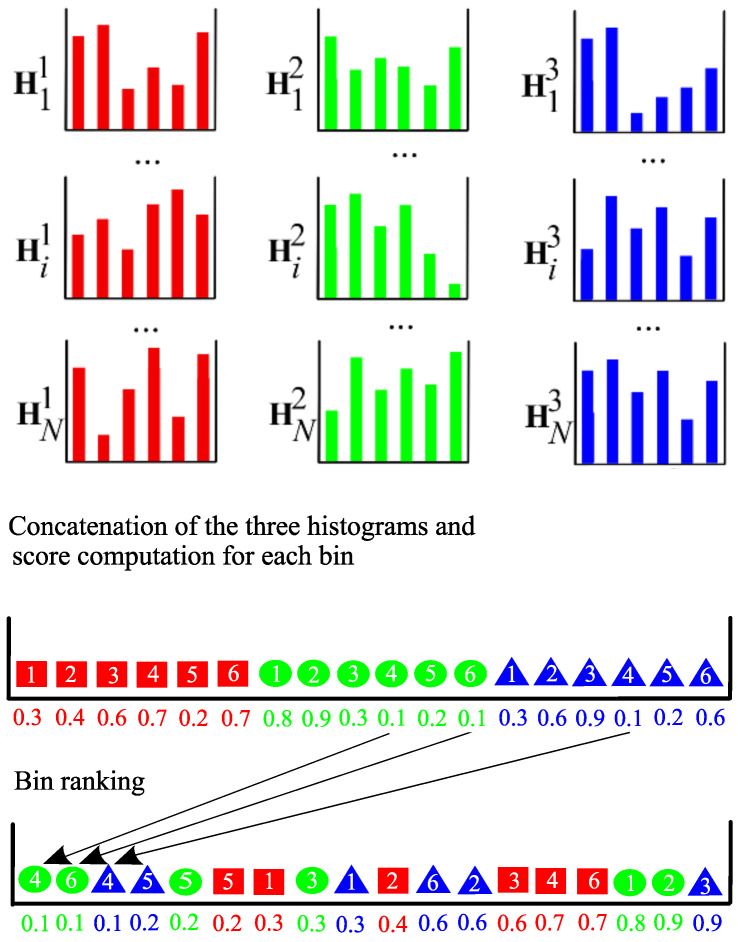
Illustration of the bin ranking.

To illustrate the proposed approach, we introduce an example with three sample histograms $H_{i}^{1}$, $H_{i}^{2}$ and $H_{i}^{3}$, i∈{1,…,N}, extracted from *N* training images, to compute the sparsity score of each bin (see Figure 3). To represent the bins of each histogram respectively, we use three symbols: a square, a circle and a triangle. We assume that each histogram has six bins which are numbered from 1 to 6. For example, the square numbered as 1 represents the first bin of histogram $H_{i}^{1}$. In our approach, the three histograms are firstly concatenated to form a feature vector with 18 features. The score associated to each bin is then computed with the sparsity score and the bins are ranked in ascending order according to their value. Note that the score value of each bin is indicated below each symbol.

Once the bin ranking strategy is applied, the dimension of the relevant bin subspace is determined. For this purpose, the relevance of candidate bin subspaces with different dimensions are evaluated. At the first step, the candidate subspace composed of the first ranked bin is considered. Then, at the second step, the candidate subspace composed of the two first ranked bins is considered and so on. Like for the MCSHS approach, the nearest neighbor classifier is considered with the L1 distance during the learning stage and the relevance of each candidate subspace is evaluated by the classification accuracy. This evaluation leads to consider a validation subset different from training images during the learning stage. This classifier operates in each candidate subspace to classify the validation images represented by their prototype bins. The selected subspace, whose dimension is $\hat{D}$, is the one which maximizes the rate of well-classified validation images denoted $R_{D}$:

(2) $$\hat{D}=\underset{1\leq D\leq Q\times \delta_{max}\times N_{S}}{\mathrm{argmax}}R_{D}.$$

During the classification stage, textures of testing images are represented in the so-selected relevant bin subspace in order to determine their class labels.

In the following section, our proposed original approach that combines a histogram selection and a bin selection for the classification task is introduced. Indeed it is clear that not all bins of the histograms selected by the MCSHS approach are meaningful for modelling the characteristics of textures. As it selects the most discriminating histograms and filter out the rest, we think that it might have some redundant bins in the selected histograms and a loss of some meaningful bins of the discarded histograms. This leads to our principal contribution that performs a combination of bin and histogram selections.

## 4. Combination of Bin and Histogram Selections

**Figure 4:**
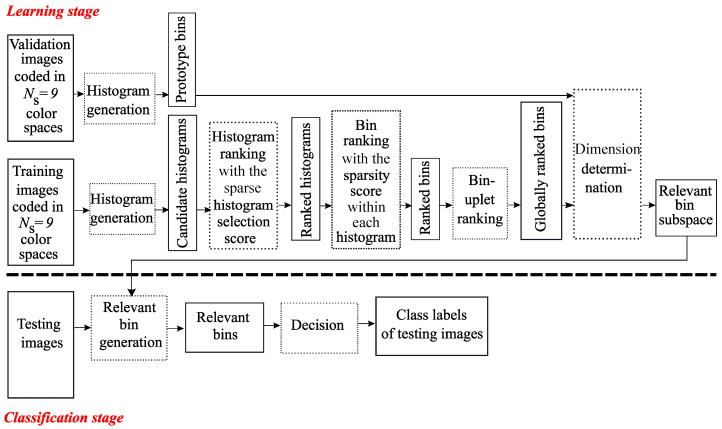
The Sparse-MCSHBS approach.

The purpose of the Multi Color Space Histogram and Bin Selection (MCSHBS) strategy is to filter out the irrelevant bins of the relevant histograms and oppositely, to find the relevant bins out of the irrelevant histograms. The flow chart of this approach is illustrated in Figure 4.

It is also an embedded selection method, where the bin ranking strategy is applied after the histogram ranking. During the learning stage, candidate histograms are generated from training images and a histogram ranking is applied by using a histogram selection score. A bin ranking strategy is then applied within each ranked histogram independently (instead of within the concatenated histogram in the MCSBS approach). Here, the SpASL-score is computed to rank the LBP histograms and the sparsity score proposed by Liu is considered to rank LBP bins. We assume that the first bin of each histogram is more relevant than the other bins. Therefore, we propose ranking at first, the group of all the first bins of each histogram in the order of the ranked histograms, then the group of all the second bins are ranked and continuously until the last bin of each histogram. The final bin ranking is a ($Q\times \delta_{max}$)-uplet vector, where *Q* is the total number of bins of each histogram and where the order of the bins in each $\delta_{max}$-uplet is based on the ranked histogram.

**Figure 5:**
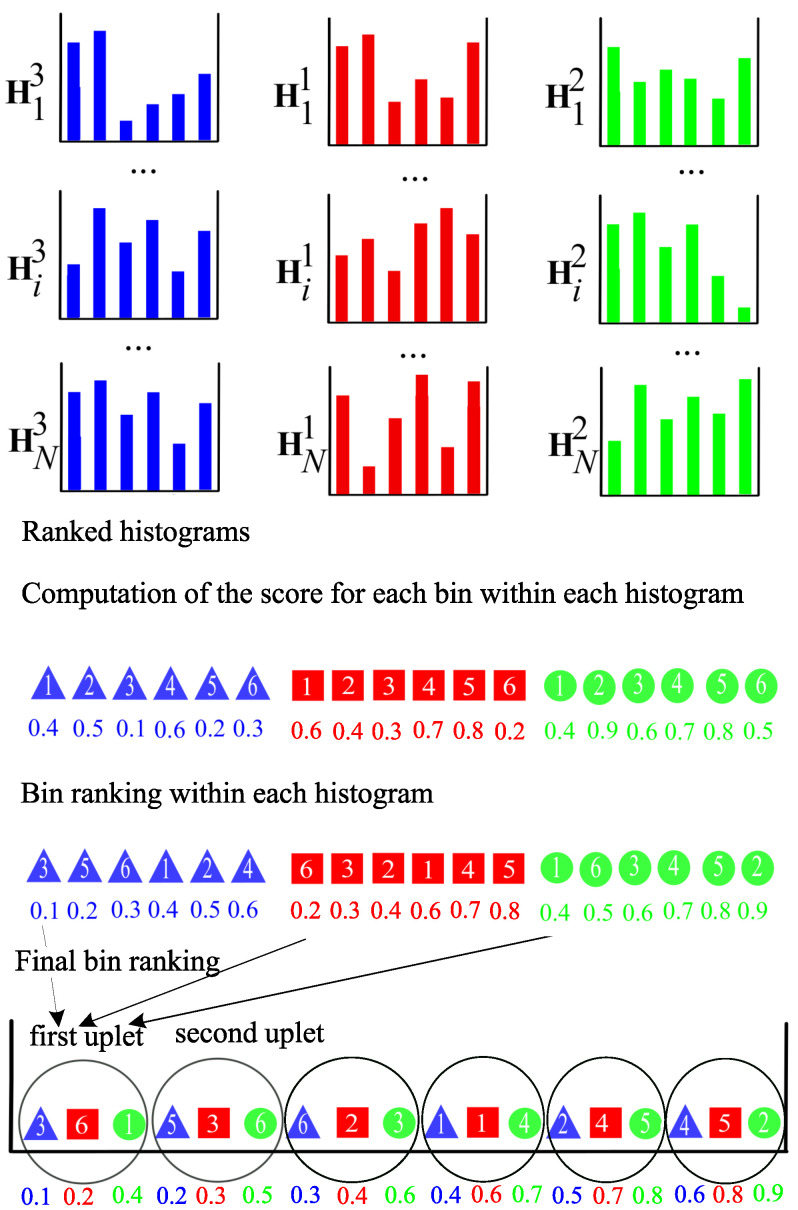
Illustration of the combination of bin and histogram ranking.

To illustrate the combination of histogram ranking and bin selection approaches, let us take the same example as in the previous section (see Figure 5). In this illustration, we assume that the histograms are ranked by a histogram selection score as $H_{i}^{3}$, $H_{i}^{1}$ and $H_{i}^{2}$. A bin ranking is then achieved within each histogram by the supervised sparsity score, whereas in the previous section, it is done within the concatenated histogram. The final bin ranking is a vector composed of the 6 triplet-bins. The first triplet is composed of the three first bins of $H_{i}^{3}$, $H_{i}^{1}$ and $H_{i}^{2}$, respectively. This procedure continues until the last triplet, which is composed of the three last bins of $H_{i}^{3}$, $H_{i}^{1}$ and $H_{i}^{2}$ respectively, is constituted.

To find the relevant subspace, the selection procedure is carried out in the same way as in Section 3.4: the relevance of candidate bin subspaces with different dimensions are evaluated thanks to the classification accuracy. The selected subspace, whose dimension is $\hat{D}$, is the one which maximizes the rate $R_{D}$ of well-classified validation images (see Equation (2)). During the classification stage, the relevant bins previously selected are computed for each testing image to determine its class label.

## 5. Experiments

In this section, we propose comparing the strategies of sparse LBP histogram selection, sparse LBP bin selection, and the combination of both in the multi color space framework (see Section 5.3). These strategies will be applied and analyzed with five image databases: NewBarktex, Outex-TC-00013, USPTex, STex and Parquet (see Section 5.1). A discussion about processing times required by the proposed selection approaches will be presented in Section 5.4.

### 5.1. Considered Color Texture Datasets

Outex-TC-00013 is composed of 68 color texture images acquired under controlled conditions by a 3-CCD digital color camera and whose size is 746×538 pixels [69]. Each of these 68 textures is split up into 20 disjoint sub-images of size 128×128. Among these 1360 sub-images, 680 are used for the training subset and the remaining 680 are considered as testing images for an holdout evaluation (This decomposition is available at http://www.outex.oulu.fi/index.php?page=classification).

USPTex set is a more recent database [70]. It contains 191 natural color textures acquired under an unknown but fixed light source. As for the previous set, these images are split up into 128×128 disjoint sub-images. Since the original image size is here 512×384 pixels, this makes a total of 12 sub-images by a texture. For our experiments, this initial dataset of 2292 sub-images is split up in order to build a training and a testing image subset: for each texture, 6 sub-images are considered for the training and the 6 others are used as testing images (This decomposition is available at http://www-lisic.univ-littoral.fr/~porebski/USPtex.zip).

The Salzburg texture image database (STex) is a large collection of 476 color texture images, whose acquisition conditions are not defined. Each of the 476 original images is split up into 16 non-overlapping 128×128 sub-images. Half of these 7616 sub-images are used for the training subset and the remaining are considered as testing images (This decomposition is available at http://www-lisic.univ-littoral.fr/~porebski/Stex.zip).

Although Outex-TC-00013, USPTex and STex sets are widely used, these image sets present a major drawback: the partitioning applied to build these three sets consists of extracting training and testing sub-images from a same original texture image. However, such a partitioning, when it is combined with a classifier such as the nearest neighbor classifier, leads to biased classification results [52]. Indeed, testing images are spatially close to training images and are thus correlated. A simple 3D color histogram is then able to reach a high classification accuracy whereas it only characterizes the color distribution within the color space and does not take into account the spatial relationships between neighboring pixels, as a color texture feature should [31]. That is the reason we propose considering two other challenging image sets built from the Parquet and the Barktex databases, respectively.

The Parquet database is composed of fourteen varieties of wood for flooring [71]. Each type of wood presents several different grades ranging from 2 to 4 which are considered as independent classes, leading to a total of 38 different classes. The main challenge of this database is that, within each type of wood, the grades are very similar to each other. Moreover, the sizes of acquired images are different and the number of samples per class varies from 6 to 8. As done in [72], six samples per class are retained and the images are centre-cropped, so that the final dimension of images ranges from 480×480 to 1300×1300 pixels. For each texture, 3 images are considered for the training and the others are used as testing images (This decomposition is available at http://www-lisic.univ-littoral.fr/~porebski/Parquet.zip). With the experimental protocol proposed by the authors, the classification accuracy reached on this challenging database does not exceed 75.1%.

The Barktex database includes six tree bark classes, with 68 images per class [73]. Even if the number of classes of this database is limited to 6, the textures of these different classes are close to each other and their discrimination is not easy. To build the NewBarktex set, a region of interest, centered on the bark and whose size is 128×128 pixels, is first defined. Then, four sub-images whose size is 64×64 pixels are extracted from each region. We thus obtain a set of 68×4=272 sub-images per class. To ensure that color texture images used for the training and the testing images are less correlated as possible, the four sub-images extracted from a same original image all belong either to the training subset or to the testing one [52]: 816 images are thus used as training images and the remaining 816 as testing images (The NewBarktex image test suite can be downloaded at https://www-lisic.univ-littoral.fr/~porebski/NewBarkTex.zip). Table 1 summarizes theses databases.

### 5.2. Performance Evaluation and Comparisons

Since the considered texture benchmark databases were built in order to apply an holdout evaluation, the classification performance is assessed by following this evaluation scheme. For this purpose, classification results reached by the proposed methods are evaluated by measuring the accuracy as the rate of well-classified testing images during the classification stage.

During this stage, the relevant histograms previously selected by one of the proposed approaches presented in Section 3 and Section 4 are computed for each testing image and compared to those of the training images in the so-selected relevant histogram or bin subspace to determine the testing image label. The purpose of this paper being to show the contribution of different LBP-based feature selection approaches, independently of the considered classifier and its parameters—such as the metric—the nearest neighbor classifier associated with the L1 distance as a similarity measure is here considered. Obviously, the classification results are expected to be improved by using more elaborated methods such as the SVM classifier for example.

As previously mentioned, the texture benchmark databases are composed of only two image subsets (training and testing images), whereas the proposed approaches need three subsets by adding validation images required by embedded selection methods. To evaluate and compare our experimental results with the same conditions of other works, which do not divide the training subset into two parts, we propose using one subset as the training subset and the second both as the validation and testing subset. During the classification stage, the supervised classifier thus uses exactly the same training subset than the one used by the compared works for determining the class labels of the testing images.

In the following, the proposed LBP-based feature selection strategies are evaluated, analyzed and compared with the results of the state-of-the-art under the same experimental protocol. Number of classes, size of images, number of images for each class, total number of images and accuracy evaluation method are the same. No changes appear about texture rotation and illumination. Therefore, comparisons exclude some other existing works that apply other protocols to the experimented databases.

### 5.3. Validation of the Proposed LBP-Based Feature Selection Strategies

Table 2 presents the results obtained with the proposed LBP-based sparse feature selection approaches, on the NewBarktex database. The single color space and multiple color space strategies are also compared, as well as the results with and without selection.

For each color space, the accuracy $R_{\hat{D}}$ estimated by the rate of well classified testing images and the dimension $\hat{D}$ of the selected feature space are presented. First, we can notice that operating a selection significantly improves the accuracy while reducing the dimensionality of the feature space. The improvement reaches on average 7.8% compared to the without selection approach.

We can also notice that even if the considered color texture database is fixed, the color space that enables reaching the best accuracy is not always the same and depends on the considered feature selection strategy: rgb (74.4%) when no selection is performed, HSV (79.5%) when a bin selection is achieved, and RGB with the histogram selection approach (81.3%) and the combination of both selections (83.7%). This confirms that the *a priori* choice of the well suited color space is not easy and so, the interest of the multi color space strategy. This table also shows that considering several color spaces significantly improves the accuracy too since an improvement of 7% is obtained compared to a single color space strategy.

We can also notice that even if the dimension of the feature space selected by the bin selection strategy is lower, the Sparse-MCSHS approach (87.3%) significantly outperforms the proposed Sparse-MCSBS approach (83.6%). This confirms the result recently obtained by Porebski et al. with the ICS-based MCSHS approach versus the MCSBS approach using Guo’s selection strategy [16]. Finally, we notice that operating a combination of bin and histogram selections (88.4%) improves the accuracy compared to a simple Sparse-MCSBS approach or a simple Sparse-MCSHS approach.

Table 3, Table 4, Table 5, Table 6 and Table 7 present the classification results obtained by our proposed approaches and those obtained by the different studies which applied a color texture classification algorithm on Outex-TC-00013, USPTex, STex, Parquet, and NewBarktex, respectively. To achieve classifier-independent comparisons, only the studies using the nearest neighbor classifier are here presented. We also propose in this paper to ignore wrapper approaches, which use the classification rate as discrimination power of feature subspaces, and so involve an important learning time [74]. We implemented on the other hand some experiments with Convolutional Neural Networks (CNN) to compare our approach with the latest popular methods. Since the considered color texture databases have few learning images, we propose using the pretrained AlexNet [75] and GoogleNet [76] networks to achieve this comparison.

The rows labelled as gray correspond to experiments that are carried out in this work whereas the other rows correspond to results published by other authors. The first column refers to the related papers and indicates the descriptors which were computed to discriminate the different color texture classes. The color spaces considered to classify the images are presented in the second column of each tables. Finally, the last column shows the obtained rate of well-classified testing images (Accuracy).

For the USPTex and STex databases, the best rate is 98.1%. These rates are obtained with our proposed approach of bin and histogram combination and are very encouraging. For Outex-TC-00013, Parquet and NewBarkTex, the results obtained with our approach get into a very satisfying position with the works of the state of the art, since they reach the second position with 95.7%, 83.3%, and 88.4%, respectively. For the Outex database, they are just behind the results obtained by the Multi Color Space Feature Selection proposed in [52] where many more different color spaces are considered. For the Parquet and NewBarkTex sets, they follow the rate of well-classified testing images reached by CNN with the pretrained GoogleNet and AlexNet network, respectively.

Table 8 presents the relative ranking of the considered approaches and the average accuracy reached over the five databases. This table shows that the combination of bin and histogram selections outperforms the simple MCSHS and MCSBS approaches: an average improvement of 0.8% compared to the MCSHS strategy and 3.1% compared to the MCSBS approach is observed. It also shows the relevance of the proposed approach with the experiments on pretrained CNN, with an average improvement of 8.2%. We can also notice that the results obtained with the proposed MCSHBS approach are more stable than those reached thanks to CNN, since they always get into the first or the second position, whatever the considered database.

As the aim of this paper is to reveal the real contribution of the proposed selection strategy as independent as possible from the impact of the classifier and LBP parameters, straightforward methods of texture representation (basic LBP configuration) and classification (1-NN) were preferred as well as texture databases without change of observation conditions. Obviously, the representation of color textures could be improved by using other configurations of LBP parameters (for instance by increasing the parameters *P* and *R*), by adding other color spaces or by considering an ensemble of several descriptors [93]. However, a high increase of the dimensionality of the original feature set could lead to a number of features much larger than the number of samples, and so to a risk of overfitting [94]. In such a case, an approach based on a preliminary feature clustering should be relevant to reduce the number of features candidate for selection.

The next section studies the impact of the proposed selection approaches on the processing times.

### 5.4. Processing Times

Moderate consumption of computation resources is really needed for applications that require fast processing and/or low memory storage such as machine vision applications operating in real time with significant production time constraints or mobile applications embedded on smartphones and tablets where the processor and the memory are limited. Supervised selection procedure aims to define a compact representation of color textures by reducing the dimensionality of the feature space during a learning stage. This offline learning stage is carried out prior to the online classification stage and, as with deep learning methods, can last from several minutes to a few days with no impact on the final application. During the online classification stage, the so defined compact representation then increases the accuracy of the classifier and decreases the computation time.

When no selection is performed, the learning stage only consists of computing the LBP histograms from the training images but the high dimension of the feature space (81×256= 20,736 in our case) leads to a high and crippling computation time for the online classification stage with a poor accuracy as illustrated in Table 2 for the NewBarktex dataset (78.2%). When a selection procedure is performed, the learning stage is more time consuming since it consists of the computation of all the available histograms from the training and the validation images, followed by a selection phase to determine the relevant feature space. As an embedded method is used during this selection phase, the dimension determination step (see Figure 1, Figure 2 and Figure 4) is the most time consuming since it requires to operate several classifications to evaluate each candidate sub-space: Equation (1) shows that $\delta_{max}\times N_{S}$ operations of classification are performed for the MCSHS approach while MCSBS and MCSHBS execute $Q\times \delta_{max}\times N_{S}$ operations (see Equation (2)). As $Q=2^{P}$ for a basic LBP descriptor, the learning execution time is strongly impacted by an increasing of the LBP parameter *P*. However, the low dimensional feature subspace determined by the selection procedure during the offline learning stage significantly reduces the online classification time.

Compared to a histogram selection approach (MCSHS), the online classification processing time reached by MCSHBS is close since the dimension of the selected feature sub-space is nearly the same: for instance, with the NewBarkTex dataset, the dimension of the selected feature sub-space is $\hat{D}=9472$ for the Sparse-MCSHS approach and $\hat{D}=$ 11,985 for Sparse-MCSHBS (see Table 2). The slight cost in processing time occurred with the Sparse-MCSHBS approach is counterbalanced by a slight gain of accuracy: 87.3% for the Sparse-MCSHS approach and 88.4% for Sparse-MCSHBS.

Compared to a bin selection approach (MCSBS), the online classification processing time required by MCSHBS is higher because the dimensionality of the feature sub-space selected with MCSBS is lower: $\hat{D}=754$ for the Sparse-MCSBS approach and $\hat{D}=$ 11,985 for Sparse-MCSHBS (see Table 2). However, classification results show that MCSHBS clearly outperforms MCSBS: the accuracy reaches 83.6% for the Sparse-MCSBS approach and 88.4% for Sparse-MCSHBS.

These results show the interest of operating a selection thanks to the proposed Sparse-MCSHBS approach.

## 6. Conclusions

In this paper, different strategies of LBP-based feature selection were developed:

- First we extended the multi color space domain with the LBP histogram selection approach using the SpASL-score to define the Sparse Multi Color Space Histogram Selection (Sparse-MCSHS).
- We then compared this approach with a new multi color space bin selection approach, also based on a sparse representation: the Sparse Multi Color Space Bin Selection (Sparse-MCSBS) approach.
- A combination of bin and histogram selections (the Sparse-MCSHBS approach) was finally developed and evaluated on several benchmark texture databases.

The obtained results has shown that the multi-color space strategy contributes to improve the classification performance, and that the proposed Sparse-MCSHBS strategy outperforms the simple MCSBS and MCSHS approaches, while getting into a satisfying position with the works of the state of the art, including CNN-based approaches. Obviously, the classification results are expected to be improved by using more recent and sophisticated methods associated with our strategy, such as the SVM classifier for example.

To operate feature selection on high-dimensional and small sample data, we currently work on an approach based on preliminary feature clustering to reduce the number of features candidate for selection. Associated with a filter method, this approach aims both to reduce the learning time and to improve the classification accuracy as well as the selection stability. Our future work will also consist in analyzing and comparing the different strategies that are proposed in the literature to simultaneously exploit the properties of several color spaces.

## Figures and Tables

**Table 1:** Summary of image databases used in experiments.

Dataset Name	Image Size	Nb of Classes	Nb of Training Images	Nb of Testing Images
Outex	128 × 128	68	680	680
USPTex	128 × 128	191	1146	1146
STex	128 × 128	476	3808	3808
Parquet	480×480 to 1300×1300	38	114	114
NewBarktex	64 × 64	6	816	816

**Table 2:** Classification results obtained by the proposed LBP-based sparse feature selection strategies in a single and multiple color spaces on the NewBarktex database (bold values represent the best rates obtained with each color space and values in boxes indicate the best rate obtained for each strategy).

Color Spaces	Without Selection		Bin Selection		Histogram Selection		Combination of Both	
	$R_{\hat{D}}$	$\hat{D}$	$R_{\hat{D}}$	$\hat{D}$	$R_{\hat{D}}$	$\hat{D}$	$R_{\hat{D}}$	$\hat{D}$
*RGB*	73.2	2304	79.0	1109	81.3	1024	**83.7**	1016
*rgb*	74.4	2304	74.4	2242	77.1	768	**77.9**	1530
$I_{1}I_{2}I_{3}$	71.7	2304	74.4	1198	79.5	1792	**80.5**	1764
*HSV*	70.5	2304	79.5	500	**81.0**	768	**81.0**	768
(wb,rg,by)	72.1	2304	77.5	836	80.6	1536	**82.1**	1524
*HLS*	70.1	2304	78.0	319	**81.0**	768	**81.0**	768
*I-HLS*	72.1	2304	72.7	1813	78.8	512	**79.1**	762
*HSI*	71.7	2304	79.2	533	**79.8**	768	**79.8**	768
$YC_{b}C_{r}$	71.6	2304	77.0	370	79.3	1792	**82.5**	1778
Average in single space	71.9	2304	76.9	991	79.8	1081	**80.8**	1186
Multiple color spaces	$\begin{array}{c} 78.2 \end{array}$	20,736	$\begin{array}{c} 83.6 \end{array}$	754	$\begin{array}{c} 87.3 \end{array}$	9472	$\begin{array}{c} 88.4 \end{array}$	11,985

**Table 3:** Comparison between the rates of well-classified image reached with the Outex-TC-00013 set and the 1-NN classifier. The accuracy values in italics were obtained with other methods that we implemented.

Features	Color Space	Accuracy
RSCCM + MCSFS [52]	28 color spaces	96.6
**EOCLBP + Sparse-MCSHBS**	**9 color spaces**	**95.7**
**EOCLBP + Sparse-MCSHS**	**9 color spaces**	**95.6**
EOCLBP + MCSHS-ICS [16]	9 color spaces	95.6
3D Color histogram [31]	*HSV*	95.4
**EOCLBP + Sparse-MCSBS**	**9 color spaces**	**95.2**
3D Color histogram [65]	*I-HLS*	94.5
Haralick features [77]	*RGB*	94.1
EOCLBP (with selection method) [18]	*RGB*	93.4
EOCLBP (with selection method) [17]	*RGB*	92.9
EOCLBP + MCSBS-Guo [16]	9 color spaces	92.9
RSCCM (with selection method) [63]	*HLS*	92.5
Between color component LBP histogram [31]	*RGB*	92.5
Quaternion-Michelson Descriptor [48]	*RGB*	91.3
Texton [78]	*RGB*	90.3
Combine color and LBP-based features [79]	*RGB*	90.2
Intensity-Color Contrast Descriptor [80]	*RGB*	89.3
DRLBP [81]	*RGB*	89.0
Autoregressive models and 3D color histogram [65]	*I-HLS*	88.9
Halftoning Local Derivative Pattern and Color Histogram [82]	*RGB*	88.2
Autoregressive models [83]	$L^{*}a^{*}b^{*}$	88.0
Within color component LBP histogram [31]	*RGB*	87.8
CWEUL LTP [84]	*RGB*	87.4
Mix color order LBP histogram [43]	*RGB*	87.1
Color angles LBP [41]	*RGB*	86.2
LBP and local color contrast [33]	*RGB*	85.3
CLBP (Completed LBP) [85]	*RGB*	84.4
Color contrast occurrence matrix [86]	*RGB*	82.6
Soft color descriptors [50]	*HSV*	81.4
Histograms of equivalent patterns [87]	*RGB*	80.9
Fuzzy aura matrices [88]	*RGB*	80.2
Pretrained AlexNet convolutional neural network	*RGB*	*78.5*
Pretrained GoogleNet convolutional neural network	*RGB*	*77.9*
Modified LBP [89]	*RGB*	67.3

RSCCM: Reduced Size Chromatic Co-occurrence Matrices, MCSFS: Multi Color Space Feature Selection, DRLBP: Dominant Rotated LBP, CWEUL LTP: Color Wavelet Elliptical Upper and Lower Local Ternary Pattern.

**Table 4:** Comparison between the rates of well-classified images reached with the USPTex database and the 1-NN classifier. The accuracy values in italics were obtained with other methods that we implemented.

Features	Color Space	Accuracy
**EOCLBP + Sparse-MCSHBS**	**9 color spaces**	**98.1**
EOCLBP + MCSHS-ASL [16]	9 color spaces	97.6
**EOCLBP + Sparse-MCSHS**	**9 color spaces**	**97.4**
EOCLBP + MCSBS-Guo [16]	9 color spaces	97.3
**EOCLBP + Sparse-MCSBS**	**9 color spaces**	**94.8**
Quaternion-Michelson Descriptor [48]	*RGB*	94.2
Halftoning Local Derivative Pattern and Color Histogram [82]	*RGB*	93.9
Quaternionic local angular binary pattern [47]	*RGB*	93.8
Pretrained GoogleNet convolutional neural network	*RGB*	*92.2*
DRLBP [81]	*RGB*	*89.4*
Color angles [41]	*RGB*	88.8
Local multi-resolution patterns [90]	Luminance	86.7
Mix color order LBP histogram [43]	*RGB*	84.2
LBP and local color contrast [33]	*RGB*	82.9
Pretrained AlexNet convolutional neural network	*RGB*	*78.3*
CLBP [85]	*RGB*	72.3
Soft color descriptors [50]	$L^{*}a^{*}b^{*}$	58.0

**Table 5:** Comparison between the rates of well-classified images reached with the STex database and the 1-NN classifier. The accuracy values in italics were obtained with other methods that we implemented.

Features	Color Space	Accuracy
**EOCLBP + Sparse-MCSHBS**	**9 color spaces**	**98.1**
**EOCLBP + Sparse-MCSHS**	**9 color spaces**	**96.7**
EOCLBP + MCSBS-Guo [16]	9 color spaces	96.7
EOCLBP + MCSHS-ASL [16]	9 color spaces	96.1
**EOCLBP + Sparse-MCSBS**	**9 color spaces**	**94.7**
Pretrained GoogleNet convolutional neural network	*RGB*	*92.9*
Pretrained AlexNet convolutional neural network	*RGB*	*90.8*
DRLBP [81]	*RGB*	*89.4*
Color contrast occurrence matrix [86]	*RGB*	76.7
Soft color descriptors [50]	$L^{*}a^{*}b^{*}$	55.3

**Table 6:** Comparison between the rates of well-classified images reached with the Parquet database and the 1-NN classifier. The accuracy values in italics were obtained with other methods that we implemented.

Features	Color Space	Accuracy
Pretrained GoogleNet convolutional neural network	*RGB*	*92.9*
**EOCLBP + Sparse-MCSHBS**	**9 color spaces**	**83.3**
**EOCLBP + Sparse-MCSHS**	**9 color spaces**	**82.5**
**EOCLBP + Sparse-MCSBS**	**9 color spaces**	**79.8**
EOCLBP + MCSBS-Guo [16]	9 color spaces	79.0
EOCLBP + MCSHS-ICS [16]	9 color spaces	75.4
EOCLBP (with selection method) [17]	*RGB*	71.9
Fisher separation criteria-based learning LBP [15]	*RGB*	*68.4*
Pretrained AlexNet convolutional neural network	*RGB*	*68.4*

**Table 7:** Comparison between the rates of well-classified images reached with the NewBarktex set and the 1-NN classifier. The accuracy values in italics were obtained with other methods that we implemented, while the underlined values indicate the results extracted from [43].

Features	Color Space	Accuracy
Pretrained AlexNet convolutional neural network	*RGB*	*90.6*
**EOCLBP + Sparse-MCSHBS**	**9 color spaces**	**88.4**
EOCLBP + MCSHS-ICS [16]	9 color spaces	88.0
EOCLBP + MCSBS-Guo [16]	9 color spaces	87.8
**EOCLBP + Sparse-MCSHS**	**9 color spaces**	**87.3**
Completed local binary count [91]	*RGB*	84.3
**EOCLBP + Sparse-MCSBS**	**9 color spaces**	**83.6**
Pretrained GoogleNet convolutional neural network	*RGB*	*82.8*
EOCLBP (with selection method) [17]	*RGB*	81.4
EOCLBP (with selection method) [18]	*RGB*	81.4
LBP and local color contrast [41]	*RGB*	80.2
Between color component LBP [37]	*RGB*	*79.9*
Light combination of LBP [92]	*HSV*	78.8
Mix color order LBP histogram [43]	*RGB*	77.7
CWEUL LTP [84]	*RGB*	76.6
RSCCM + MCSFS [52]	20 color spaces	75.9
CLBP [85]	*RGB*	*72.8*
Color angles [33]	*RGB*	71.0
DRLBP [81]	*RGB*	*61.4*
Color histograms [31]	*RGB*	*58.6*

**Table 8:** Relative ranking of the considered approaches and average accuracy reached over the five databases.

Approach	Relative Ranking					Average Accuracy
	Outex	USPTex	STex	Parquet	NewBarkTex	
EOCLBP + Sparse-MCSHBS	1	1	1	2	2	92.7
EOCLBP + Sparse-MCSHS	2	2	2	3	3	91.9
EOCLBP + Sparse-MCSBS	3	3	3	4	4	89.6
Pretrained GoogleNet CNN	5	4	4	1	5	87.7
Pretrained AlexNet CNN	4	5	5	5	1	81.3
